# Cord compression defined by MRI is the driving factor behind the decision to operate in Degenerative Cervical Myelopathy despite poor correlation with disease severity

**DOI:** 10.1371/journal.pone.0226020

**Published:** 2019-12-26

**Authors:** Bryn Hilton, Jennifer Tempest-Mitchell, Benjamin M. Davies, Jibin Francis, Richard J. Mannion, Rikin Trivedi, Ivan Timofeev, John R. Crawford, Douglas Hay, Rodney J. Laing, Peter J. Hutchinson, Mark R. N. Kotter

**Affiliations:** 1 School of Clinical Medicine, University of Cambridge, Cambridge, England, United Kingdom; 2 Division of Neurosurgery, Department of Clinical Neurosciences, Addenbrooke's Hospital and University of Cambridge, Cambridge Biomedical Campus, Cambridge, England, United Kingdom; Cleveland Clinic, UNITED STATES

## Abstract

**Objectives:**

The mainstay treatment for Degenerative Cervical Myelopathy (DCM) is surgical decompression. Not all cases, however, are suitable for surgery. Recent international guidelines advise surgery for moderate to severe disease as well as progressive mild disease. The goal of this study was to examine the factors in current practice that drive the decision to operate in DCM.

**Study design:**

Retrospective cohort study.

**Methods:**

1 year of cervical spine MRI scans (N = 1123) were reviewed to identify patients with DCM with sufficient clinical documentation (N = 39). Variables at surgical assessment were recorded: age, sex, clinical signs and symptoms of DCM, disease severity, and quantitative MRI measures of cord compression. Bivariate correlations were used to compare each variable with the decision to offer the patient an operation. Subsequent multivariable analysis incorporated all significant bivariate correlations.

**Results:**

Of the 39 patients identified, 25 (64%) were offered an operation. The decision to operate was significantly associated with narrower non-pathological canal and cord diameters as well as cord compression ratio, explaining 50% of the variance. In a multivariable model, only cord compression ratio was significant (p = 0.017). Examination findings, symptoms, functional disability, disease severity, disease progression, and demographic factors were all non-significant.

**Conclusions:**

Cord compression emerged as the main factor in surgical decision-making prior to the publication of recent guidelines. Newly identified predictors of post-operative outcome were not significantly associated with decision to operate.

## Introduction

Degenerative Cervical Myelopathy (DCM) is a progressive condition characterized by degenerative changes in the cervical spine leading to chronic spinal cord compression. Pathological changes include osteophytosis, intervertebral disc bulging, and ligament ossification and hypertrophy leading to static and dynamic injury to the spinal cord [1]. DCM is the commonest cause of spinal cord dysfunction with an estimated minimum incidence and prevalence of 41 and 605 per 1,000,000 respectively in North America. The actual prevalence is likely to be much higher given under and misdiagnosis is common [2]. Estimates based on imaging series could put the prevalence as high as 5% in over 40 year olds [3,4]. In an aging population, DCM looks to become an increasing healthcare burden.

DCM is diagnosed through a combination of signs and symptoms consistent with a clinical diagnosis of myelopathy alongside an MRI scan showing the aforementioned pathological changes. Myelopathic features include: loss of manual dexterity, imbalance and falls, urinary and bowel dysfunction, hyper-reflexia, and limb weakness and spasticity [5]. DCM progresses in a step-wise or continuous manner and leads to significant reductions in a patient’s quality of life [6,7]. Given the non-specific and insidious presentation of DCM, assessment may prove challenging and significant delays have been noted with regards to diagnosis and treatment [8,9].

The mainstay of DCM management is surgery, but the optimal timing of surgery can be challenging, as the rate of disease progression is highly variable and cannot currently be predicted [2]. Current evidence does not demonstrate an ‘optimal’ surgical approach, and it should instead be tailored to the individual case depending on factors such as the location of the pathology, number of cervical levels affected, and the baseline cervical sagittal alignment [10].

In the largest prospective series of DCM patients undergoing surgery, Tetreault et al. found the following factors predict a better post-operative functional status: younger age, milder pre-operative myelopathy, non-smoker, fewer co-morbidities, non-impaired gait, shorter pre-operative symptom duration [11]. Other studies have highlighted the importance of short time duration between symptom onset and surgery in maximising patients’ postoperative function [12,13,14]. So far, pre-operative MRI factors have not shown to add further predictive power but do influence surgeons with regards to operative approach [15,16]. In their latest update on outcome prediction, Tetreault et al (2018) demonstrate that symptom duration and baseline disease severity remain the strongest and most consistent indicators [17].

In order to standardise management of DCM patients, an international group involving multiple stakeholders recently proposed the first set of guidelines [18]. These guidelines are based around an internationally accepted 18-point scale for the severity of DCM, the Modified Japanese Orthopaedic Association (mJOA) score. The guidelines recommend:

1. Surgery for cases of moderate (mJOA 12–14) or severe (mJOA <12) DCM.
2. Surgery or supervised non-operative treatment for mild (mJOA 15–17) DCM.
3. Surgery is strongly recommended in the case of progressive deterioration.
4. No surgery for asymptomatic cord compression.
5. Surgery or close follow-up for non-myelopathic patients with cord compression with radiculopathy symptoms.

Prior to the publication of the guidelines, the decision to operate was largely left to the treating surgeon. The present study therefore sought to establish which clinical or radiological features were used to guide treatment decisions prior to the release of these guidelines, whether these included features that were shown to be predictive of post-operative outcomes, and to provide a reference against which adoption of guidelines could be assessed. This is the first study to examine this critical moment in DCM management and to compare past practice with international best practice guidelines.

## Methods

**Fig 1:**
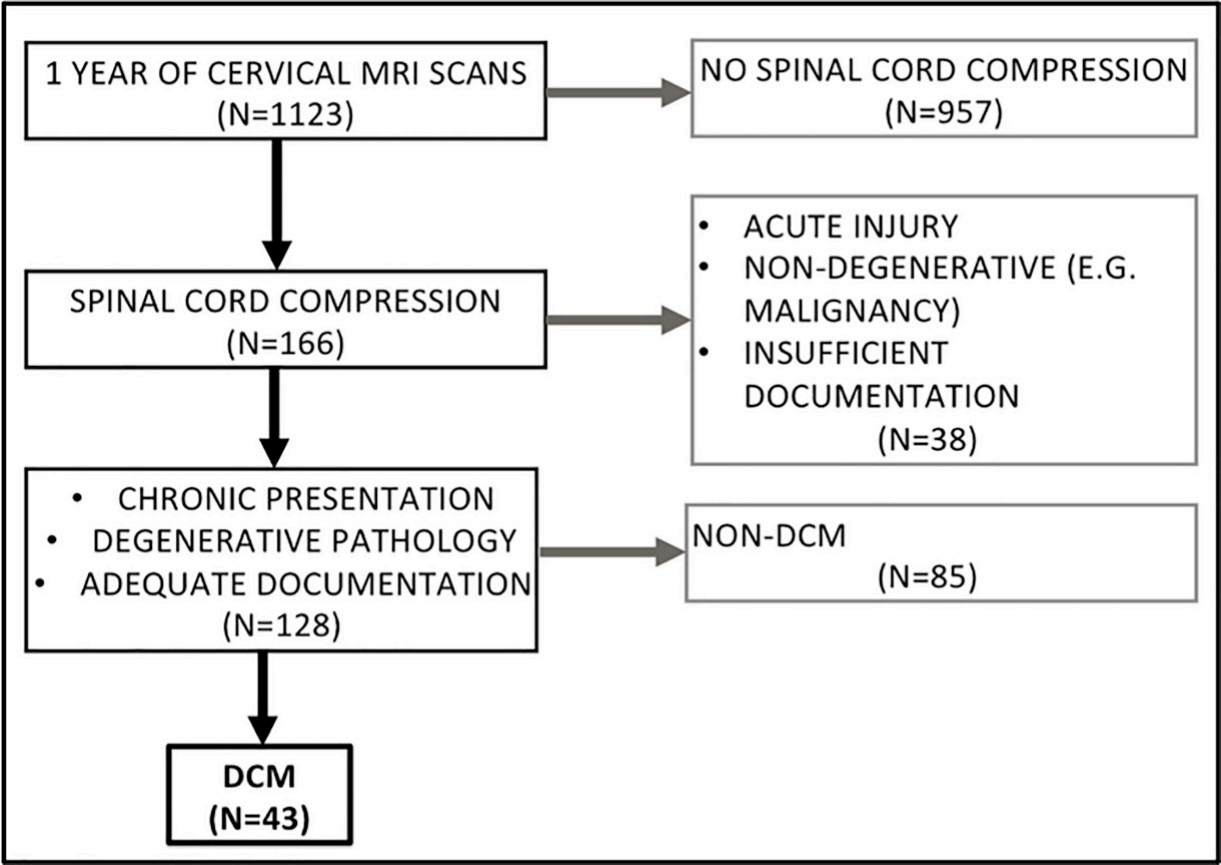
PRISMA flow diagram depicting cohort formation methodology.

This was a retrospective, observational study based on a cohort of DCM patients (N = 39) identified from screening one year’s worth of cervical MRI scans (N = 1123) at a tertiary neurosciences centre. The case notes of patients with radiological cord compression were screened for a clinical diagnosis of DCM [8, 9] (Fig 1). All demographic and clinical data was collected from case notes as per the prior protocol. The inferred modified Japanese Orthopaedic Assessment (i-mJOA) was developed and used as a validated proxy for mJOA to assess disease severity as described [17]. The ‘decision to operate’ was defined by the clinician offering the patient an operation, regardless of intended surgical approach and if the patient decided to go ahead with surgery or not.

Quantitative MRI measurements commonly used in DCM literature were collected as per previous methodology [19]. Measurements used in this study were: maximum canal compromise (MCC) [20], maximum spinal cord compression (MSCC) [19], spinal canal occupation ratio (SCOR)[21], compression ratio (CR) [22], normal canal diameter (NCaD), and normal cord diameter (NCoD). NCaD and NCoD were defined as the average anterior-posterior diameter of the spinal canal and cord respectively at the first non-degenerative spinal level above and below the diseased segment.

Statistical analysis was carried out using SPSS V22 (Chicago, IL, USA). The following statistical tests were applied depending on variable types involved in the analysis. Chi-squared test of homogeneity was used for and Fisher’s exact were applied for the relationship between clinical features (dichotomous, present or absent) and the decision to operate. Pearson’s correlations were used for i-mJOA (ordinal) relative to the decision to operate. Bivariate correlations followed by binomial logistic regression were applied for quantitative MRI measures (continuous) relative to the decision to operate.

All data was collected as part of a localised audit and quality improvement project. The work was formally registered and permission acquired in 2016 as a Service Evaluation project entitled “Degenerative cervical myelopathy: correlating clinical encounters with disease progression and radiological appearances using retrospective functional assessment”, Department of Clinical Neurosciences, Addenbrooke’s Hospital, University of Cambridge. All patient data was fully anonymised prior to collection and there was no active patient participation in this study.

## Results

### Cohort summary

43 cases of DCM were initially identified in this cohort. The average age at time of symptom onset was 61.4±13.9 years and the majority of patients (28, 65%) were male. Except for one patient, all cases were assessed by a spine surgeon. 48% (20/42) of these cases were new DCM while 52% (22/42) were recurrent DCM.

The overall times between symptom onset and surgical assessment for new and recurrent cases of DCM were 17.7±16.0 and 9.5±9.0 months respectively. Of the new cases of DCM, 45% (9/20) received an operation, 35% (7/20) received a follow-up appointment, and 20% (4/20) were discharged. Of the recurrent cases of DCM, 50% (11/22) received an operation, 23% (5/22) received a follow-up appointment, and 27% (6/22) were discharged. The details of 3 surgical assessments were not included beyond this descriptive cohort analysis. The reasons for this were: one case also suffered from severe Parkinson’s disease thus blurring symptomatic origin, one case did not attend their appointment, and one case’s assessment notes were inaccessible. 77% (30/39) of cases had sufficient documentation available to track disease progression prior to arrival at surgical assessment.

### Correction between MRI measurements and clinical features of DCM

In order to investigate how objective measures of spinal cord compression impact on functional deficits, every clinical feature recorded at surgical assessment was examined relative to every MRI measurement (Table 1). A narrower NCaD was associated with corticospinal motor deficits (p = 0.04), clonus (p = 0.001), upper limb paraesthesia (p = 0.027), and lower limb paraesthesia (p = 0.014). A narrower NCoD and SCOR each showed one significant association with clonus (p = 0.02) and limb pain (p = 0.017) respectively. MCC, MSCC, and CR were not significantly associated with the presence of any clinical feature of DCM.

**Table 1:** The relationships between MRI measurements and clinical features at surgical assessment.

Clinical feature	MCC	MSCC	NCaD	NCoD	SCOR	CR
Paraesthesia (upper limb)	0.97	0.66	0.03*	0.27	0.23	0.55
Weakness (upper limb)	0.43	0.88	0.84	0.40	0.17	0.32
Paraesthesia (lower limb)	0.94	0.29	0.01*	0.20	0.11	0.60
Weakness (lower limb)	0.88	0.47	0.44	0.87	0.30	0.92
Limb pain	0.21	0.46	0.36	0.41	0.02*	0.74
Neck pain/stiffness	0.29	0.53	0.46	0.78	0.40	0.94
Sphincter dysfunction	0.35	0.84	0.62	0.38	0.49	0.34
Instability	0.24	0.09	0.84	0.66	0.61	0.61
Falls	0.96	0.45	0.65	0.91	0.52	0.61
Corticospinal motor deficits	0.41	0.77	0.04*	0.44	0.08	0.77
Hyper-reflexia	0.99	0.92	0.22	0.18	0.93	0.66
Positive hoffmann	0.99	0.27	0.89	0.48	0.32	0.68
Upgoing plantars	0.31	0.51	0.07	0.11	0.95	0.35
Clonus	0.61	0.26	0.001*	0.02*	0.10	0.46
Unstable gait	0.56	0.91	0.55	0.51	0.93	0.63
Upper limb motor i-mJOA	0.19	0.13	0.89	0.94	0.79	0.19
Lower limb motor i-mJOA	0.37	0.59	0.52	0.37	0.66	0.60
Sensory i-mJOA	0.60	0.95	0.99	0.44	0.25	0.78
Sphincter i-mJOA	0.59	0.52	0.91	0.59	0.52	0.52
Total i-mJOA	0.94	0.91	0.64	0.67	0.73	0.34

* = significant at 95%.

The relationship between MRI features and an imputed version of the main clinical tool for assessing dysfunction in DCM (i-mJOA) was examined based on total scores and broken down by subcategory (Table 1). Neither subcategories of i-mJOA nor total i-mJOA score were significantly related to any of the MRI measures. Taken together, morphological features of spinal cord compression did not correlate well with clinical findings.

### Relationship between clinical features and decision to operate

Current guidelines base the decision to operate mainly on clinical presentation of DCM. We therefore investigated the individual relationships between clinical features and the decision to operate prior to publication of the guidelines (Table 2). No clinical feature reached statistical significance relative to the decision to operate. Age (p = 0.19) and sex (p = 0.45) were also both found to have no significant influence on clinical management.

**Table 2:** The relationships between clinical features and the decision to operate ordered by ascending p-value. Number of DCM cases the feature was recorded as present in as a percentage of total cases.

Clinical Feature	Cases present (%)	p-value
Paraesthesia (lower limb)	38	0.12
Clonus	16	0.12
Limb pain	53	0.22
Lower limb spasticity	3	0.25
Subjective weakness (lower limb)	33	0.30
Hyper-reflexia	80	0.34
Paraesthesia (upper limb)	65	0.48
Unstable gait	28	0.63
Subjective weakness (upper limb)	38	0.68
Falls	10	0.75
Sphincter dysfunction	5	0.83
Subjective imbalance	35	0.85
Positive Hoffmann reflex	47	0.89
Objective corticospinal motor deficits	40	0.90
Neck pain/stiffness	35	0.97
Upgoing plantars	47	0.98

The relationship between the decision to operate and the i-mJOA score, total and broken down by subcategory was investigated (Table 3). No subcategory of i-mJOA or total i-mJOA score was significantly related to the decision to operate.

**Table 3:** The relationships between i-mJOA scores, change in i-mJOA scores, and the decision to operate.

i-mJOA category	Average +/- SD	p-value
Upper limb motor	4.1±0.9	0.52
Lower limb motor	5.7±1.5	0.16
Sensory	2.1±0.7	0.91
Sphincter	2.9±0.3	0.79
Total	14.8±2.5	0.24
Upper limb motor change	-0.2±0.7	0.61
Lower limb motor change	-0.3±0.8	0.07
Sensory change	-0.2±0.8	0.88
Sphincter change	0±0.3	0.61
Total change	-0.8±2.0	0.20
Total deterioration*	n/a	0.28

*The relationship between if the patient had deteriorated at all, regardless of degree, with the decision to operate.

Progression of DCM reflected in deterioration of mJOA scores are now thought to provide a strong indication for surgery. Change in i-mJOA between the first available assessment (primary or secondary) and pre-operative assessment by a surgeon were therefore calculated and examined relative to the decision to operate (Table 3). Deterioration in i-mJOA, subcategory or total, was not significantly associated with an increased likelihood of being offered an operation.

### Relationship between MRI measurements and the decision to operate

To investigate whether imaging parameters influence treatment, the relationships between individual MRI measurements and the decision to operate were studied (Table 4). Bivariate correlations showed significant relationship between decision to operate and NCaD (p = 0.024), NCoD (p = 0.019), and CR (p<0.001). Nagelkerke R^2^ = 0.495. Binomial logistic regression using these three significant variables showed only compression ratio as a significant predictor of decision to operate (p = 0.017).

**Table 4:** The relationships between MRI measurements and the decision to operate.

MRI measurements	p-value
MCC	0.50
MSCC	0.53
NCaD	0.02*
NCoD	0.02*
SCOR	0.97
CR	<0.001**

* = significant at 95%

** = significant at 99%.

### Relationship between decision to operate and international guidelines

Finally, we examined the decision to operate in this cohort relative to the new international guidance outlines above [5]. 29/39 cases (74%) were managed in line with these guidelines. Of the remaining 10 cases: 4 had severe myelopathy (i-mJOA <12) and were not offered surgery, 4 had moderate myelopathy (i-mJOA 12–14) and were not offered surgery, and 2 had asymptomatic cord compression (i-mJOA 18) and were offered surgery.

## Discussion

Overall, this study found that the only factor significantly associated with offering a patient an operation was the compression ratio of the cervical spinal cord. Features such as disease severity (assessed through i-mJOA functional scoring) and disease progression were not significantly associated with offering a patient an operation. This finding suggests that surgeons are basing their interventions on images and not on the patients in front of them. Although compression ratio is not routinely undertaken in a clinical setting, it can feasibly act as a proxy for how compressed the cord appears upon examining sagittal MRI slices. Indeed, compression ratio alone appeared to capture 50% of the variability in the decision to offer a patient an operation. It has been previously observed through a survey of 689 international spinal professionals that a strong belief exists that MRI is a valuable prognostic tool in DCM. Our study affirms this observation in routine clinical practice.

It is known that degenerative changes in the spine are increasingly common with age [19]. Whilst the natural history of asymptomatic cervical spinal cord compressions secondary to degenerative changes is not fully understood, the risk of developing myelopathy in the short-term appears low [23]. In one of the few studies to prospectively observe asymptomatic cord compression, Bednarik et al identified that abnormal electrophysiology, hyperintense lesions on T2-weighted MRI, and radiculopathic symptoms [24] predicted progression to myelopathy. The amount of pre-operative spinal cord compression has not been shown to be good indicator of post-operative outcomes. As has been previously demonstrated, the degree of spinal cord compression on MRI correlates poorly with clinical signs of myelopathy [25–27]. Our study further supports this point. The degree of spinal cord compression on MRI also correlates poorly with symptomatology and patients’ functional disability [21,28]. Patients with minimal cord compression may experience debilitating symptomatology and those with substantial cord compression may experience little to no symptoms at all. Therefore, amount of cord compression on MRI is not a suitable factor to hold such influence over the decision to operate in DCM.

Imaging techniques to predict response to surgery remain a significant research focus, with an emphasis on new techniques and technology. A potential issue blurring the correlation between MRI appearance and symptomatology is that the vast majority of cervical MRI scans are static. They are taken with the patient supine in a cervical position that may not correspond to their normal erect cervical alignment. A study found that the cervical canal narrowed at C5/6 and C6/7 when a force was applied cranially that simulated body weight whilst in the supine position [29]. Dynamic MRI, taken in flexed and extended neck positions, may yield a more accurate representation of cord compression occurring in a patient’s day-to-day life. Dynamic MRI has shown improved visualisation of significant canal stenosis and T2 hyperintense lesions on the spinal cord [30–32]. Furthermore, dynamic injury though lesions underestimated on static MRI may cause repetitive neurovascular trauma to the spinal cord and lead to significant symptomatology and disability for patients. Unfortunately, this type of imaging is not widely available for general use. Other forms of advanced imaging such as DTI and PET also show promise but are again currently a long way from routine clinical use [33–35].

A systematic literature review carried out this year found that the two factors most predictive of post-operative outcome in DCM are duration of symptoms prior to surgery and disease severity [36]. Current international guidance proposes that surgery be offered to all patients with moderate DCM or progressive disease. No mention of imaging characteristics is mentioned in these guidelines for offering an operation. We observed 74% concordance with these guidelines. However, it appears that degree of spinal cord compression is driving this decision-making, rather than patients’ functional disability or disease progression. This approach is problematic as may offer operations for patients who are asymptomatic and put them under undue risk for prophylactic treatment of disease that may never progress. Furthermore, static MRI imaging may underestimate the degree of cord and nerve impingement present in an erect position and may thus mislead surgeons as to the most problematic cervical levels to target surgically.

With regards to the discrepancy between best practice guidelines and observed practice, it is important to note that the guidelines were published after the cohort used for this study. However, the difficulties in changing established surgical practice have been well described [37]. Two particular issues that Meshikhes proposes seem especially pertinent here: individual clinical experiences and publication bias. Firstly, if cord compression has been driving DCM operative decisions for years, surgeons will inevitably see patients with significant cord compression who make good improvement post-operatively. Such cases were observed in our cohort. Current knowledge suggests that this is likely coincidence rather than a link between MRI findings and post-operative outcomes. However, such clinical observations may distort surgeons’ perceptions of appropriate indications for surgery. Furthermore, these observations may be passed down to trainee surgeons before they have even established their own clinical observations. And secondly, given the heavy focus on surgery as the central research interest in DCM [38], there may well exist a reluctance to publish data showing poor improvement post-operatively or stable patients not requiring surgery. Such issues must be confronted in order to align current practice with evidence-based best practice guidelines.

Ultimately, surgery is the current mainstay treatment for DCM, by anterior or posterior approach, and should be based on clinical assessment, not imaging findings. Current surgical management can therefore be easily improved by more evidence-based stratification of operative candidates: moderate and severe myelopathy, any progressive disease, and consideration in radiculopathic patients with cord compression.

## Limitations

This study was conducted in a single tertiary neurosciences centre. Thus, it is possible that decision-making may be more homogeneous than would be observed in an inter-centre study. However, as the decision to operate was not MDT-guided, there is a strong case to believe that surgeons were freely expressing their individual clinical judgments when deciding whether to operate or not. Furthermore, although smaller than recent international studies, the sample size used in this study should adequately display correlations in decision-making tendencies. Future multi-centre studies of greater sample size would provide further insight into factors currently influencing the decision to operate in DCM.

## Conclusion

This work demonstrates that the decision to operate is multi-faceted. However, despite its poor representation of disease severity and response to treatment, cord compression is a major factor in surgical decision-making. Whilst imaging features are required to make a diagnosis, they are not recommended for use in selecting surgical candidates. Current international best practice guidelines recommend surgery for: moderate and severe myelopathy, any progressive disease, and consideration in radiculopathic patients with cord compression. Further work is needed to evaluate the integration of these guidelines into wider clinical practice.

## Supporting information

S1 File Supplementary data. Excel file containing raw data collected in this research and used for analysis.
(XLSX)
